# Fear of COVID 19 Infection Across Different Cohorts: A Scoping Review

**DOI:** 10.3389/fpsyt.2021.708430

**Published:** 2021-09-07

**Authors:** Shalini Quadros, Shalini Garg, Rupesh Ranjan, Guruprasad Vijayasarathi, Mohammed A. Mamun

**Affiliations:** ^1^Department of Occupational Therapy, Manipal College of Health Professions, Manipal Academy of Higher Education, Manipal, India; ^2^Achutha Menon Centre for Health Science Studies, Sree Chitra Tirunal Institute of Medical Sciences and Technology, Thiruvananthapuram, India; ^3^National Homoeopathy Research Institute in Mental Health, Kerala Central Council for Research in Homeopathy, Ministry of Ayurveda, Yoga and Naturopathy, Unani, Siddha and Homeopathy Government of India, Kottayam, India; ^4^CHINTA Research Bangladesh, Dhaka, Bangladesh; ^5^Department of Public Health and Informatics, Jahangirnagar University, Dhaka, Bangladesh

**Keywords:** COVID-19 and psychological impact, fear of COVID-19, Covid-19 panic, fear of infection, mental health, scoping review, COVID-19 fear, prevalence and risk factors

## Abstract

**Background:** Psychological stressors like panic, fear, phobia, etc., are being substantially reported during the COVID-19 outbreak. In the prior outbreaks, fear of being infected was reported as the prominent suicide stressor. Therefore, fear of infection has become a concern in the context of the COVID-19 pandemic because it worsens emotion, cognition, and behavioral responses. Understanding the extent of fear of COVID-19 infection in various cohorts would aid in gauging the mental health services, which was a remedy in the present review.

**Methods:** Adhering to Arksey and O'Malley's framework for conducting a scoping review, a systematic search was performed in the month of September 2020 in several databases, including Scopus, PubMed, Web of Science, etc. Considering the inclusion criteria, a total of 14 articles were included in the present review.

**Results:** All of the included studies were conducted via online platforms, whereas all but one of the studies were cross-sectional in nature (including a mixed-method study, and a comparative study). Most of the studies were conducted among the general population (*n* = 12), within March and May 2020 (*n* = 9), from Asian countries (*n* = 7), and considered a self-developed item for fear of COVID-19 assessment (*n* = 8; whereas the Fear of COVID-19 Scale was used in 6-studies). The prevalence of fear of COVID-19 was reported to be 18.1–45.2%, although no cutoff point or criteria was mentioned for such a prevalence estimation of the Fear of COVID-19 Scale. However, females, younger adults, urban residents, divorcees, healthcare workers, those in quarantine settings, those in suspicion of being infected, and those with mental health problems, etc., were found to be at an increased risk of COVID-19 fear.

**Conclusions:** Being one of the first reviews in this context, the findings are anticipated to be helpful to predict the possible solutions for reducing fear of COVID-19 and facilitate further studies on strategies of how to alleviate such a stressful situation.

## Introduction

COVID-19 is a disease which is infectious in nature and caused by the newly introduced virus named SARS-CoV-2. Individuals infected with this virus generally present the symptoms of fever, body ache, cough, nasal congestion, loss of taste and smell in milder cases, and chest pain and breathing difficulties in severe cases. Because of its rapid transmission globally, the WHO declared it a pandemic in early 2020. However, more than 152 million people worldwide are infected with the virus, whereas more than 3.19 million deaths are recorded as of 5 May 2021.

The COVID-19 pandemic has not only resulted in physical conditions, social, psychological, and economic consequences are also being observed globally; whereas the combined role of the alteration of normal life leads people to suffer from a higher degree of mental health problems, including fear of infection, uncertainty, stress, anxiety disorders, sleep problems, mood disorders, suicidality etc. (1–3). Public health interventions have increased the feelings of discomfort and economic loss, which mediates the mental instabilities more harshly (4). Additionally, changes in daily lives and restriction of movement such as working from home, schooling, restricted play for children, and restricted contact with friends and family have led people to suffer from higher stress and anxiety levels (4, 5). As per the Centre for Disease Control and Prevention (5), fear and stress related to the COVID-19 have led to symptomatology, including change in sleep and eating patterns, worsening of premorbid psychiatric conditions, and increased use of substances (e.g., alcohol, tobacco, drugs), which are frequently alleged for mental health burdens (6).

During the COVID-19 pandemic, suicide mortality rate increment is being observed as consistent with the prior pandemics (7). However, four major types of suicide stressors have been identified in the prior pandemics, whereas fear of being infected was regarded as the prominent suicide factor followed by social isolation, disruption of normal life, and burden of long-term illness (8, 9). In the context of the COVID-19 pandemic, fear of COVID-19 infection is also reported as the main suicide mediating factor (1, 7, 10, 11). Studies have identified various domains of fear related to the fear of COVID-19 infection, such as fear of oneself or their family members getting infected, fear of having economic losses and being unemployed, or fear of avoidance behaviors toward gaining knowledge about the pandemic or fear of making decisions on showing or not showing actions like whether to visit parents or not, whether to look for information on death rates or not, etc. (12, 13). The deaths caused due to the pandemic have been enormous, inflicting a sense of fear among people. However, people worrying about being infected with the virus is being regarded as the fear of COVID-19 despite the diversity of fears related to the pandemic (6, 14), which are also investigated in this review.

Fear is defined as “a basic, intense emotion aroused by the detection of imminent threat, involving an immediate alarm reaction that mobilizes the organism by triggering a set of physiological changes” (15). During the current pandemic, the fear was about either being infected or infecting others (14, 16). Understanding fear is an important part of individual and community well-being as it influences how an individual participates in daily occupations. Occupational participation is the ability of an individual to participate in occupations of their choice, and is the satisfaction given within the boundaries of the culture (17). The fear might also affect how people react to control guidelines required as preventive measures that aid in the overall outcome of the disease transmission in the community (18). The fear of COVID-19 infection has led many individuals to abort their participation in social activity (19), which even leads to suicidal attempts in extreme cases (7, 10, 11). Due to the guidelines enforced to control the pandemic, people are deprived of participation in various occupations such as participating in social gatherings, traveling, and so on, which means an imposed lack of opportunities to participate in various occupations for reasons not under the control of individuals, and this occupational deprivation leads to ill-health (20).

As the pandemic gradually extended across the globe, the effects have been experienced in the population at large and certain groups within. While stress-related to fear of being infected, loss of lives and livelihoods are affecting the entire population, certain groups like older adults, healthcare workers, caregivers, migrants, women and children exposed to abuse, people with pre-existing mental health conditions, and people with disabilities, who are already vulnerable need to be given special mental health attention (21–23). As fear may help in explaining several of these consequences, it is important to understand what creates this fear and what the predictors are (3).

Longstanding fear may stimulate the behavioral immune system, which usually leads to aversive emotions, cognition, and behavioral responses (24). Though these aversive behaviors initially help in staying away from illnesses (25), prolonged exposure to fear may lead to emotional and distress-related disorders (26). Understanding the extent of this fear of infection in various populations would aid in gauging the extent of community mental health services needed for different population groups to meet the needs of the people who experience the psychological responses that have occurred due to the fear of infection. In addition, meaningful occupations are necessary for experiencing well-being (27), and hence an understanding of the kind of occupations that are affected due to the fear of COVID-19 may help in thinking of innovative ways through which occupational participation can be facilitated in individuals.

Scoping reviews allow us to map evidence and synthesize knowledge on a topic following a systematic approach and identify main concepts, theories, and knowledge gaps (28). This review can help map the extent of the evidence on a given topic and identify gaps in the literature to help determine the direction of future research (28, 29). In this scoping review, therefore, we attempt to map evidence on the fear of COVID-19 across various groups of the population to seek factors that affect fear and the consequences of fear related to the COVID-19 pandemic in the short-term and long-term health of individuals. The present review is anticipated to entail the extent of this problem in different populations, identify various groups at increased risk, and identify gaps in the literature to focus our future research on this global crisis.

## Methods

The initial framework was proposed for scoping reviews by Arksey and O'Malley (30), later on, it was extended by other researchers (28). However, as it is widely used, Arksey and O'Malley's framework was used in this scoping review. Accordingly, the methodology is described in the following stages:

- **Stage 1**. Identifying the research question,
- **Stage 2**. Searching for relevant studies,
- **Stage 3**. Selection of studies,
- **Stage 4**. Charting of data,
- **Stage 5**. Collating, summarizing, and reporting the results

### Stage 1: Identifying the Research Question

All five reviewers contributed to refining the research question through frequent discussions and pilot searching. Based on the discussion, the reviewers came up with the following research question: “What is the available literature on the effects of fear of COVID-19 across different populations?” Therefore, the objectives of the current scoping review were decided as follows: (i) to review the literature available on the existence of fear of COVID-19 in children, adolescents, adults, and older adults, (ii) to map the literature available on the factors related to the fear of COVID-19 that influence occupational participation of children, adolescents, adults, and older adults (if there is available literature across these cohorts).

### Stage 2: Searching for Relevant Studies

Having specific criteria for searching the relevant articles is a requirement to answer the question framed for any scoping review (30). Hence, following the inclusion criteria were decided on for the scoping review by all the reviewers.

The databases Scopus, PubMed/MEDLINE, ProQuest, Web of Science, Journal Citation Reports, CINAHL Plus etc., were accessed for retrieving relevant articles. The search strategy included 4 types of keywords: (i) outcome of interest (fear OR scare OR terror OR dread); AND (ii) exposure (COVID-19 OR lockdown OR quarantine OR isolation); AND (iii) cohort (children OR kid OR adolescent OR teenager OR youth OR adults OR elderly OR geriatric); AND (iv) Occupation (activities OR activities of daily living OR self-care OR work OR housework OR leisure OR sleep OR social participation OR education). Searches were performed in combination with the keywords in the month of September 2020. However, the inclusion and exclusion criteria for including studies in the present review are presented in Table 1.

**Table 1:** Inclusion/exclusion criteria for including studies in the present review.

	**Inclusion criteria**	**Exclusion criteria**
Population	Studies on participants with or without being positive for COVID-19	Studies conducted on healthcare workers and individuals with specific illnesses
Intervention	Literature focusing on fear of COVID-19	Studies conducted on general mental status
Comparison	Studies comparing the extent of fear across various cohorts such as children, adolescents, adults, and older adults	Studies comparing fear between individuals with and without illnesses
Outcome	Fear of COVID-19	Not related to fear of COVID-19
Others	Studies published in the English language from December 2019 to August 2020 and studies with full-text being available by journal or pre-print server	Publications of letters to editors, correspondence, point of views, ideas, opinions, studies exclusively done on healthcare workers, studies on development and validation of the fear scales

### Stage 3: Selection of Studies

SG and RR reviewed titles and abstracts to identify the first set of articles relevant to our research question. Any kind of disagreement was resolved after consulting with GV. If any disagreement still existed, then the article titles were still included in the initial list. The reviewers GV and SQ blindly reviewed the abstracts included in the first list, and in case of any disagreement, reviewers SG and MM were approached for finalizing the decision. The full text of all the articles included in the first list was searched for. All possible ethical ways (such as contacting the author, requesting the library) were tried to obtain the full text of any article that was not available freely on search. The final version of full-text articles was blindly reviewed by SG, RR, GV, and SQ independently. If the reviewers came up with any discrepancies, the matter was discussed and finalized with consultation from MM.

**Figure 1:**
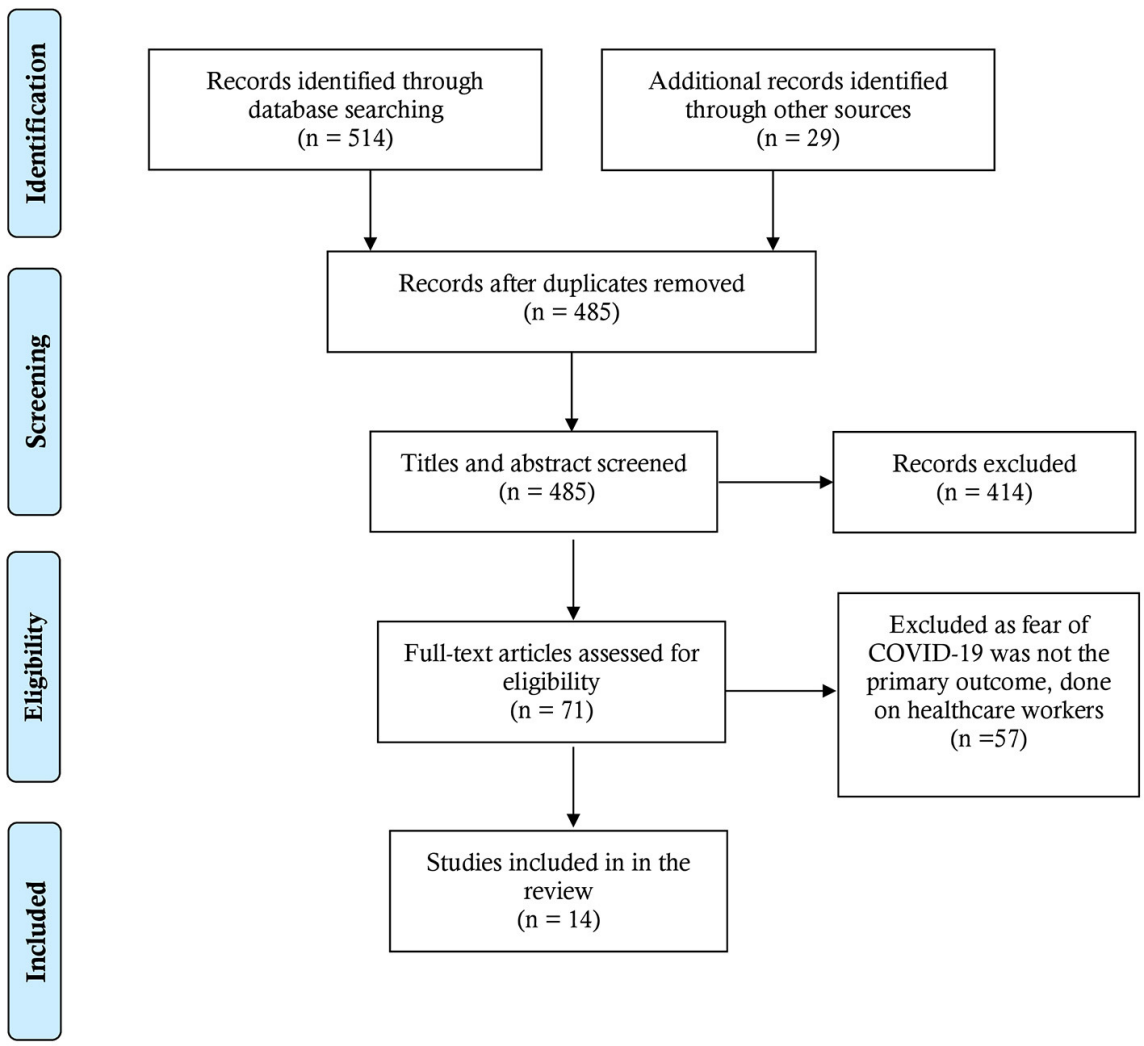
PRISMA flow-chart of this scoping review (31).

Figure 1 illustrates the search done while conducting this scoping review that has adhered to the PRISMA-ScR (Preferred Reporting Items for Systematic Reviews and Meta-Analysis extension for Scoping Reviews) guidelines (29).

### Stage 4: Charting of Data

Data extraction criteria of the present scoping review were determined, and a self-designed data extraction form was designed by consultation of the reviewers before aiding the process. The included articles were reviewed in full-text and summarized under the headings as mentioned in Table 2. The reviewers independently summarized the findings and cross-checked with each other, and the final outline is presented in the Table. In respect to providing answers to the study question, data extraction from the included studies were as follows: a bibliography of the study (author's name and publication date), data collection time, study design, sample size, country of the study conducted, specific group and mean age, participants being COVID-19 infected or suspected, and outcome(s).

**Table 2:** Descriptions of the included studies in the present review.

**References**	**Data collection time**	**Study design**	**Sample size**	**Country**	**Specific group and mean age**	**Being COVID-19 infected or suspected**	**Assessment tool**	**Outcome(s)**
Andrade et al. (32)	May 12 to 24, 2020	Cross-sectional	1,743	Brazil	General people; 30.61 ± 8.68 years	11 were COVID-19 infected, 96 were suspected of having the COVID-19 infection	Brazilian Fear of COVID-19 Scale	Fear of COVID-19 infection scores were lower in males with occupational risk of contamination, whereas females and younger individuals were at greater risk of fear of COVID-19
Bakioǧlu et al. (33)	March to April 2020	Cross-sectional	960	Turkey	General people; 29.74 ± 9.64 years	Details of whether COVID-19 infected or under quarantine is not specified	Turkish Fear of COVID-19 Scale	Fear of COVID-19 infection scores were higher in participants being women and having chronic illnesses; fear of COVID-19 was correlated with intolerance of uncertainty, depression, anxiety, and stress
Bäuerle et al. (34)	March 10 to May 5, 2020	Cross-sectional	15,037	Germany	General people; ≥18 years	Details of whether COVID-19 infected or under quarantine is not specified	Self-developed single item (response 1 to 7)	Fear of COVID-19 infection scores were lower in males, whereas younger individuals were at greater risk of having a fear of COVID-19
Broche-Pérez et al. (35)	April 4 to May 27 2020	Cross-sectional	772	Cuba	General people; 36 ± 14.61 years	Details of whether COVID-19 infected is not specified, but participants were not under quarantine	Spanish (Cuban) Fear of COVID-19 Scale	Fear of COVID-19 infection scores were more severe in female genders
Doshi et al. (18)	April 25 to 26, 2020	Cross-sectional	1,499	India	General people; 20–60+ years	Details of whether COVID-19 infected or under quarantine is not specified	Fear of COVID-19 Scale	Females, married status, lower educational status, and being a health care worker had higher levels of fear of COVID-19 infection
Fitzpatrick et al. (36)	March 23 to 30, 2020	Cross-sectional	10,368	US	General people; ≥18 years	Details of whether COVID-19 infected or under quarantine is not specified	Self-developed single item (response 0 to 10)	Women, Hispanics, Asians, families with children under 18, and foreign-born participants had higher levels of subjective fear and worry related to COVID-19
Haddad et al. (37)	April 3 to 18, 2020	Cross-sectional	407	Lebanon	General people; 30.59 ± 10.10 years	Details of whether covid-19 infected is not specified, but participants were either quarantined or confined	Self-developed single item (response 1 to 5)	Fear and anxiety were more and more than half of the participants were abiding by home quarantine/confinement.
Islam et al. (38)	May 5 to 15, 2020	Cross-sectional	340	Bangladesh	General people; 26.23 ± 6.39 years	Details of whether covid-19 infected or under quarantine are not specified.	Self-developed single item (response 1 to 6)	Fear of COVID-19 infection (i.e., self and/or family member(s), and/or relatives), hampering scheduled study plan and future career, and financial difficulties leading to human stress.
Jaspal et al. (39)	Not reported	Cross-sectional	411	UK	General people; 48.85 ± 15.38 years	Details of whether COVID-19 infected is not specified, but participants 10% were under quarantine	Fear of COVID-19 Scale	Muslims demonstrated higher levels of fear than Christians
Li et al. (40)	December 2019 to April 2020	Longitudinal	555	China	College students; 19.6 ± 3.4 years	Details of whether COVID-19 infected is not specified, but participants were confined due to lockdown	Self-developed single item (response 0 to 10)	Gender, negative mood, depression, anxiety, etc. were correlated with fear of COVID-19 infection
Mertens et al. (3)	Not reported	Cross-sectional	439	Global	General people; 26.0 ± 11.7 years	Participants were not infected with covid-19, but details about being quarantined are not specified	Fear of the Coronavirus Questionnaire	Male gender, health anxiety, the risk for loved ones, and looking up additional information (i.e., through regular media and social media) were independent predictors for fear of COVID-19
Nicomedes and Avila (19)	Not reported	Mixed method cross-sectional	538	Philippines	General people; 23.82 (range 13–67) years	Participants were exposed to COVID-19 infection, but details related to quarantine are not specified	Self-developed qualitative item	Fear of COVID-19 infection was one of the themes identified in this qualitative study
Saurabh and Ranjan (41)	Not reported	Comparative cross-sectional (with 131 non-quarantined)	121	India	Children and Adolescents; 9–18 years	Participants were in primary contact with COVID-19 infected person and were under quarantine just before the study	Self-developed items	Quarantined children and adolescents experienced greater psychological distresses (e.g., worry, helplessness, fear related to COVID-19) than non-quarantined
Šljivo et al. (42)	April 7 to 12, 2020	Cross-sectional	1,201	Bosnia and Herzegovina	General people; 30.57 ± 11.26 years	Participants were not covid-19 infected, and details related to quarantine are not specified	Fear of COVID-19 Scale	Being older, female, living in an urban area, having moderate to severe depressive symptoms were significant independent predictors for developing a fear of COVID-19

## Results

### Description of the Included Studies

#### General Description of the Studies

A total of 14 studies were included in this review, where eleven studies were reported to have been conducted between March and May 2020. Out of 14 studies included herein, all were conducted via online platforms. Thirteen studies were cross-sectional in nature (including one mixed-method and one comparative study), whereas one was a longitudinal study. The sample size of the studies varied from 121 to 15037. Most of the studies were conducted among the general adult population, a study was conducted among children and adolescents (mean age = 15.4 years), and another study was conducted among college students (mean age = 19.6 years). In respect to the geographical location, most of the included studies (*n* = 7) were conducted in Asian countries [i.e., Bangladesh (38), China (40); India (18, 41); Lebanon (37); Philippines (19); and Turkey (33)], where no studies belonged to African and Australian continents.

#### Measures Used in the Studies

In most of the studies, fear of COVID-19 was assessed using a self-developed item (*n* = 7), whereas the Fear of COVID-19 Scale was used in a total of 6-studies. Note, the Fear of COVID-19 Scale assesses the fear that exists specifically related to the COVID-19 infection (14). Three studies used a validated version of the Fear of COVID-19 Scale in the languages of Brazilian (32), Turkish (33), and Spanish (Cuban) (35) and the original scale was used in three studies. Self-developed items were used to assess different kinds of variables like socio-demographic, health problems, COVID-19 related issues, etc.; whereas psychological problems (e.g., depression, anxiety, stress, intolerance of uncertainty etc.) were assessed using respective psychometric tools.

### Prevalence of Fear of COVID-19

The prevalence of COVID-19 fear assessing by the Fear of COVID-19 Scale was reported in two studies (18, 42). Doshi et al. (18), in the Indian study, estimated that 45.2% of subjects feared about COVID-19, although no cutoff point was mentioned for such a prevalence estimation. Similarly, an 18.1% prevalence of strong fear associated with COVID-19 was also reported by Šljivo et al. (42) without any clarification about the used cutoff score. In addition, a 5-point Likert scale-based ten-item was used to determine higher fear of COVID-19 in another study (44.8%), although the cutoff point was not reported (37).

### Factors Associated With Fear of COVID-19

Gender was reported as the most consistent predictor, with more women experiencing moderate to high fear levels of COVID-19 (18, 32, 33, 35, 36, 42). Age was also significantly associated with fear of COVID-19 as per a few studies, and it was generally found that older people were less scared of the disease (32, 42). Another interesting predictor of COVID-19 fear was an occupational risk, that is, being engaged in high COVID-19 risk professions such as healthcare professionals. In one study, it was associated with higher levels of COVID-19 fear (18), although men working in higher-risk occupations were found to have low levels of COVID-19 fear as per Andrade et al.'s study (32). Again, participants being in quarantine settings or with a suspicion of being infected with the virus showed elevated levels of COVID-19 fear (32, 41). In addition, being Muslim compared to Christian (39), having maladaptive eating behaviors (37), even the COVID-19 information sources (3, 39) were identified as increasing the risk of higher fear of COVID-19.

### Distribution of Fear of COVID-19 Across Cohorts

#### Fear of COVID-19 Among Children and Adolescents

Only one study was specifically conducted among children and adolescents from 9 to 18 years of age who were in quarantine, which was a comparative study in nature (with non-quarantined ones) (41). The COVID-19 fear was assessed using a pre-formed questionnaire, whereas quarantined children and adolescents were found to experience greater psychological distress (e.g., worry, helplessness, fear related to COVID-19) than non-quarantined ones.

#### Fear of COVID-19 Among Students

The only longitudinal study was conducted to determine the mental health status of the college students in considering “before” and “during” confinement periods, where the fear of COVID-19 severity was reported higher after they were confined (40). In the students being confined, during the follow-up assessment, gender, negative mood, depression, anxiety, etc., were found to be correlating factors of COVID-19 fear (40).

#### Fear of COVID-19 Among Adults

Several studies were conducted among adults (3, 18, 19, 32–38, 42). Fitzpatrick et al. (36), in an online survey on people above 18 years of age, found that the fear of COVID-19 varied from region to region, and women especially were more vulnerable to COVID-19 fear. Similarly, females were reported to be at an increased risk of COVID-19 fear by another study (18), whereas being divorced and having a low educational background were other factors associated with fear of COVID-19.

#### Fear of COVID-19 Among Older Adults and Elderly

No studies were specifically conducted focusing on older adult cohort, although studies among general people found heterogeneous findings regarding elder age-related distribution of COVID-19 fear. That is, Doshi et al. (18) observed the participants being elderly (more than 60 years) were at lower risk of fear of COVID-19 compared to the groups of younger age. Whereas, 65.0 and 65.3% of the participants with 65–74 years and more than 75 years old, respectively, had reported at risk of elevated COVID-19-related fear, which was 55.2% for the age group of 18–24 years (34).

#### Fear of COVID-19 Among General Population

Of the included studies, 12- were conducted among the general population (3, 18, 19, 32–39, 42). Hence, the population categories in terms of the extent of fear among children, adolescents, adults, and the elderly cannot be differentiated. The subgroup of the general population is reported as it included study participants, including children, adolescents, adults, and the geriatric population. Analysis of the specific cohorts from this sub-group may not convey any meaning as such, but the findings for the sub-group of the general population may help in making some sense of fear-related factors in various age groups. All the studies have identified many factors contributing to higher levels of fear of COVID-19. Specifically, a higher level of COVID-19 fear was observed in female participants (32, 33, 35), but one of the studies also identified that the males were at a higher risk of fear of COVID-19 infection (3). Similarly, the contributing role of age in the participants' COVID-19 fear levels was reported in many studies (18, 32, 34, 42). Whereas, the only longitudinal study (40) observed an increment trend of COVID-19 fear compared while the participants were confined as similarly observed in another study (37). In addition, the only qualitative study found the panic responses of the general population related to the COVID-19 pandemic, in which fear was one of the themes identified (19). Two studies identified that fear of COVID-19 infection was higher in people exposed more to media for information on COVID-19 compared to others (3, 39).

## Discussion

To the best of the author's knowledge, no prior review was conducted summing up the COVID-19 fear, like other mental health problems related to the pandemic. Therefore, the present scoping review being very first in this context, is anticipated to help gauge the extent of fear of COVID-19 in various populations such as children, adolescents, adults, and older adults. Thus, the findings reported herein may help to predict the possible reasons for fear of COVID-19 and also facilitate further research on strategies to alleviate such a situation.

Based on the present findings, it is evident that female genders are at higher risk of fear of COVID-19 infection (18, 32, 33, 35, 36, 42). The reason for gender-based heterogeneity in contributing fear of COVID-19 can be explained by the prior studies, whereas males were reported as having irresponsible attitudes toward the COVID-19 pandemic, which dramatically decreases their consciousness about the potential infection of the virus (43, 44). In addition, studies have reported that women experience more fear and anxiety (45, 46); however, they are more resilient and deal well in difficult times (33). Besides this, some aging people are at lower risk of COVID-19 fear (32, 34), which can be attributed to these adults' resilience and coping strategies (47, 48). Furthermore, older adults' successful involvement in community and family-related activities, favorable physical and mental health would have contributed to their resilience resulting in less fear in the older population in comparison with the younger populations – which is suggested to focus on policy actions.

One of the studies by Jaspal et al. (39) has identified the association between religion and levels of fear of COVID-19 and found that Muslims demonstrated higher levels of fear than Christians. This was associated with their sources of information and other stressors. COVID-19 fear was also associated with eating disorders in a sample in Lebanon (37). Some studies have also tried to explore the pathways through which several factors affect mental health. Fear of COVID-19 was found to be associated with job insecurity and depression, insomnia etc. (49, 50), whereas Mahmud et al. (51) found a mediating role of fear of COVID-19 infection between depression related to COVID-19 and career anxiety. In addition, there seems to be an association between the information people look up through various sources and fear (3, 39). Intense fear can influence decision-making capacities, emotional regulation capacities, and relationship issues in people; which is suspected to be considered in policy practice (52).

The extent of COVID-19 fear may influence an individual's participation in daily life activities and follow up with the guidelines introduced by the government. Most of the included studies examined anxiety and depression and other mental health problems by established assessment tools, although it is fear of COVID-19 which was assessed by the developed tool, Fear of COVID-19 Scale, in a total of 6-studies. Fear of COVID-19 Scale has good psychometric properties and is being widely used to measure fear related to the COVID-19 pandemic (53). Therefore, it is recommended to be considered in future research as fear is a key source of anxiety and distress-related mental health problems (49). Besides, all of the studies were carried out by online surveys, which increases the risk of potential for selection bias. These studies might not represent the true population due to the over-representation of certain groups like women and younger adults. In addition, all studies except one have a cross-sectional design; therefore, causal relationships reported by the individual studies cannot be inferred. Although studies included in this review were from various parts of the world, generalizations should be made more cautiously.

Another factor that needs to be considered here is the occupation of the participants included. Though the current review did not include studies done specifically on health care workers, it is unsure whether the included studies herein were conducted on the general population, including healthcare workers. Therefore, it is suggested to provide more information on study participants to make a better comparison across studies because healthcare professionals are found to have higher fear of COVID-19 due to higher exposure in healthcare settings, leading to suicide (54, 55). Another drawback is that none of the included studies reported any information related to the participants' mental health disorders, which may attribute fear of COVID-19 to a great extent (56).

Most of the studies included in the current review assessed the fear during the initial phases of the pandemic, that is, from March to May 2020, when the whole world was facing the first wave of the COVID-19 pandemic. Associations existed between seeking information on COVID-19 related information on social media and its fear (57), which could have been more during the initial stages of the pandemic. The studies included have assessed fear using self-developed items that are not standardized, and hence the reliability of the results obtained can be questionable. Nevertheless, a few studies have used the fear of COVID-19 scale to assess the extensively used fear and evaluate fear explicitly related to COVID-19. Furthermore, most of the studies included are cross-sectional in nature the temporal relationship between the COVID-19 infection and fear cannot be inferred. In addition, the quality of the included study (e.g., time length of the survey, the validity of the measurement tool, and how statistical significance of the association factors) could be added to construct the concrete of the study finding interpretation, which may limit the findings. However, being one of the first approaches summarizing the fear of COVID-19, the limitations of this scoping review are supposed to be addressed in further reviews.

## Concluding Remarks

The COVID-19 pandemic has helped draw attention to the issues that affect physical and mental health. Studies that further our understanding of these issues are just gathering up, where the present review adds baseline information to this context. However, it is important to consider the long-term consequences of the pandemic as this pandemic unfolds at different speeds in various parts of the world. Low-and middle-income countries that are struggling with resources to fight the disease and dealing with a huge burden of poverty and hunger have gotten more than they can handle. Fear of COVID-19 has induced unwanted reactions in people from running away from their livelihoods to commit suicide to an increase in domestic violence. It is important to understand what people are afraid of during these pandemics, the associated factors, and how this can be alleviated or at least be managed. Long-term effects of COVID-19 fear can be dangerous and grow into adverse effects later in life. This review illustrated that people are afraid of getting infected either for themselves or their loved ones. Mostly women, younger adults, and information gathered through media are associated with the fear of COVID-19. It is important to keep in mind that several programs try to induce fear to improve compliance for protective behavior. However, studies have demonstrated that this strategy does not work (58). Therefore, these messages should be informative rather than being fear-inducing. This review underlines the fact that further studies are required investigating fear of COVID, as the world is moving into the second year of the COVID-19 pandemic with more aggressive infections sweeping many areas.

Being one of the first reviews in this context, the findings are anticipated to be helpful to predict the possible solutions for reducing fear of COVID-19 and facilitate further studies on strategies of how to alleviate such a stressful situation. The vulnerable population and associated factors of COVID fear identified through this review may help policymakers develop appropriate strategies to handle the current crisis of long-term effects of the pandemic on people's mental health.

## Author Contributions

SQ and SG have contributed to the initial pilot search, planning the methodology, and writing. RR and GV have contributed to planning the methodology, data gathering, and reviewing the draft. MM has contributed to planning the methodology and writing. SQ and MM addressed the reviewers' comments and revised the manuscript critically. All authors approved the final version of the manuscript.

## Conflict of Interest

The authors declare that the research was conducted in the absence of any commercial or financial relationships that could be construed as a potential conflict of interest.

## Publisher's Note

All claims expressed in this article are solely those of the authors and do not necessarily represent those of their affiliated organizations, or those of the publisher, the editors and the reviewers. Any product that may be evaluated in this article, or claim that may be made by its manufacturer, is not guaranteed or endorsed by the publisher.
